# Environmental legacy contributes to the resilience of methane consumption in a laboratory microcosm system

**DOI:** 10.1038/s41598-018-27168-9

**Published:** 2018-06-11

**Authors:** Sascha M. B. Krause, Marion Meima-Franke, Annelies J. Veraart, Gaidi Ren, Adrian Ho, Paul L. E. Bodelier

**Affiliations:** 1 Department of Microbiology, University of Washington, Seattle, WA USA; 2 Department of Microbial Ecology, Netherlands Institute of Ecology (NIOO-KNAW), Wageningen, The Netherlands; 3 Department of Aquatic Ecology and Environmental Biology, Institute for Water and Wetland Research, Radboud University, Nijmegen, The Netherlands; 4 Institute of Agricultural Sciences and Environments, Jiangsu Academy of Agricultural Sciences, Nanjing, China; 5 State Key Laboratory of Soil and Sustainable Agriculture, Institute of Soil Science, Chinese Academy of Sciences, Nanjing, China; 6 Institute of Microbiology, Leibniz Universität Hannover, Herrenhäuserstr. 2, Hannover, Germany

## Abstract

The increase of extreme drought and precipitation events due to climate change will alter microbial processes. Perturbation experiments demonstrated that microbes are sensitive to environmental alterations. However, only little is known on the legacy effects in microbial systems. Here, we designed a laboratory microcosm experiment using aerobic methane-consuming communities as a model system to test basic principles of microbial resilience and the role of changes in biomass and the presence of non-methanotrophic microbes in this process. We focused on enrichments from soil, sediment, and water reflecting communities with different legacy with respect to exposure to drought. Recovery rates, a recently proposed early warning indicator of a critical transition, were utilized as a measure to detect resilience loss of methane consumption during a series of dry/wet cycle perturbations. We observed a slowed recovery of enrichments originating from water samples, which suggests that the community’s legacy with a perturbation is a contributing factor for the resilience of microbial functioning.

## Introduction

Microbial communities are crucial components of every ecosystem, and are important drivers of global biogeochemistry^1^. Their significance is now widely established with an increased interest to understand the world’s microbiomes^2,3^. A pressing research question is the quantification and prediction of the response of microbial processes to the increase of extreme weather phenomena as well as human impacts on natural systems.

Environmental history has been identified as an determining factor for current microbial communities and functioning^4,5^. In so-called legacy effects, past biotic and abiotic conditions persist through time even if the environment is altered, which affects the response to changing environmental conditions in present microbial communities^6,7^. Therefore, it is reasonable to assume that microbial communities from environments without prior exposure to a specific change in environmental conditions may only have limited capacity to respond to such a perturbation^8^.

Resilience is a way to quantify such responses and is broadly defined into engineering resilience, which simply refers to the recovery over time, and ecological resilience, which measures the amount of disturbance necessary to move a system to an alternative stable state^9^. Both processes are connected in the concept of critical slowing down that has been introduced to biology only recently^10^. It suggests that the rate of recovery from multiple small scale disturbances (i.e. engineering resilience) can be used as a measure to a tipping point in biological systems at which the system switches into another stable state (i.e. ecological resilience)^10,11^. Note that slowed recovery is not only a sign of an impending catastrophic shift, but can also signal potential decreasing stability in systems without alternative stable states^12^, whereas rapid regime shifts and chaotic bifurcations can also occur without slowing of recovery rates^13,14^.

The existence of this novel indicator in biological systems has been shown in previous studies. For instance, Dakos and Bascompte^15^ detected indications for critical slowing down using the structure of 79 mutualistic networks from ecological communities. In another study Veraart and colleagues^16^ used cyanobacteria as an example to demonstrate critical slowing down before an induced transition to a tipping point. Hence, slowed recovery may represent a suitable indicator with great promises to rank the response to perturbation of complex microbial systems.

However, measuring specific microbial responses in complex environmental communities is still a challenging task. Alternatively, laboratory model systems represent a simplified approach that enables controlled manipulations and detailed analysis of individual species responses and feedbacks.

Aerobic methane-oxidizing bacteria (methanotrophs) are a key microbial group that catalyzes the degradation of a major greenhouse gas and is thus of great importance for the global climate and methane budget^17,18^. Previous studies using methanotrophs demonstrated a high recovery rate from experimental disturbances such as dry-wet cycles^8^, desiccation and heat stress^19^, nitrogen pulse at different methane source strengths^20^, and have been shown to recolonize disturbed habitats^21^ suggesting that these organisms have a high capacity to survive and persist a range of environmental conditions. In addition, laboratory microcosms and field experiments using stable isotope probing techniques have shown that in the presence of methane as main carbon source, co-occurring non-methanotrophic communities are not random^22,23^, allowing to reduce the total microbial community to functionally relevant, potentially interacting non-methanotrophic taxa. Hence, aerobic methane consumption represents an ideal model system to study basic principles of microbial resilience with a defined subset of interacting microbial communities.

In this study, we hypothesized that the functionally relevant methanotrophic communities originating from soil (always dry), sediment (periodic dry) and water (never dry), i.e. different legacies in exposure to dry-wet cycles, would become increasingly vulnerable to perturbations due to loss of “ecological resilience”. To test whether biomass (expressed as abundance) and changes in the total non-methanotrophic community by the perturbations are the driver underlying a loss of resilience, we designed a laboratory microcosm setup using diluted and undiluted enrichments of methanotrophs and the associated total microbial community. We focused on recovery as an indicator that can be used to detect resilience loss in important microbial ecosystem processes.

## Results

### Methane consumption rates

**Figure 1:**
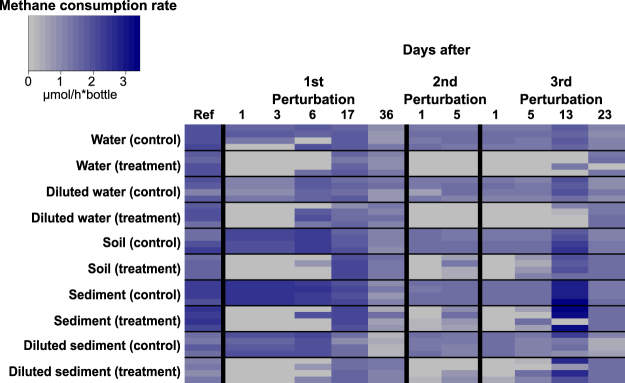
Methane consumption rates of individual microcosm enrichments at different time points during the course of the experiment (n = 4 for each group). The first column (Ref) depicts methane consumption rates from microcosms after two weeks of pre-incubation and before the first dry/wet cycle perturbation.

First, we observed that no methane consumption could be measured from any environment one day after each dry/wet cycle perturbation (Fig. 1). Second, most replicates from different environments resumed activity after seventeen days following the first perturbation (Fig. 1). After the second and third perturbation cycle, we observed a pattern in which samples from different environments resumed activity already after five days, except for the water samples (Fig. 1). We did not find any apparent trend with enrichments from diluted samples (Fig. 1).

### Recovery of methane consumption after each dry/wet cycle perturbation

**Figure 2:**
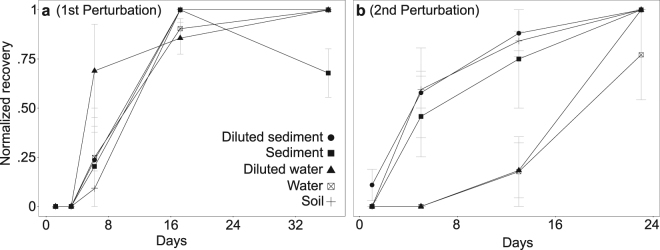
Normalized recovery of methane consumption rates after the first dry/wet cycle perturbation (**a**) and the third dry/wet cycle perturbation (**b**), (mean ± s.d; *n* = 4 for each setup). A detailed description can be found in Material and Methods.

We then calculated recovery from perturbation as a metric to describe resilience loss of an important microbial process (Supplementary Information Table S1). We show that the lag phase in all environments and dilutions after the first perturbation (Fig. 2a) disappeared from the soil and sediment samples but increased in the water samples after the third perturbation (Fig. 2b). Again, no apparent trend was observed with enrichments from diluted samples (Figs 1 and 2).

### Community composition after a series of dry/wet cycle perturbation

**Figure 3:**
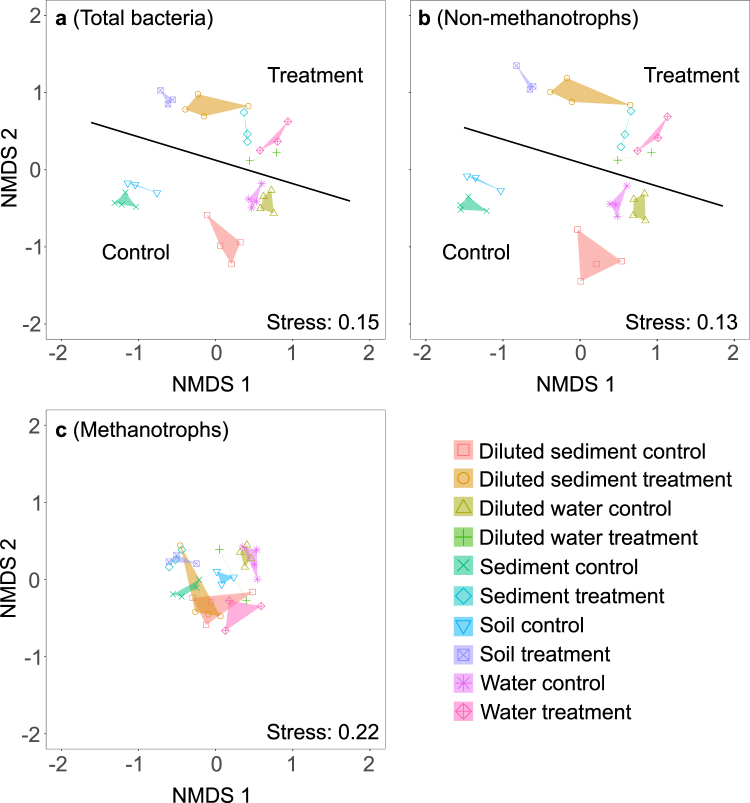
Nonmetric multidimensional scaling analysis showing the community composition of the total and methanotrophic community (**a**), the fraction of non-methanotrophic bacteria (**b**), and the fraction methanotrophic bacteria (**c**) derived from the standardized 16S rDNA-based sequencing data (treated water samples n = 3, treated diluted water samples n = 2, treated and control soil samples n = 3, treated sediment samples n = 3, all remaining n = 4).

We used Illumina 16S rDNA sequencing to evaluate whether changes in the total bacterial methanotrophic community structure were linked to the observed differences of resilience in methane consumption from different environments. The microbial community composition in all samples from different environments had significantly different community structures (ANOSIM R: 0.864, *P* < 0.001; Fig. 3a). We identified a separation based on environment and treatment (Fig. 3a). We then partitioned the data set into methanotrophs and non-methanotrophs. Intriguingly, the non-methanotrophic part of the community contributed strongest to the separation of different environments (Fig. 3b) while the methanotrophic community was more similar among samples (Fig. 3c). An analysis of the diversity parameters richness, evenness, and Shannon index did reveal any obvious trends (Supplementary Information Table S2).

**Figure 4:**
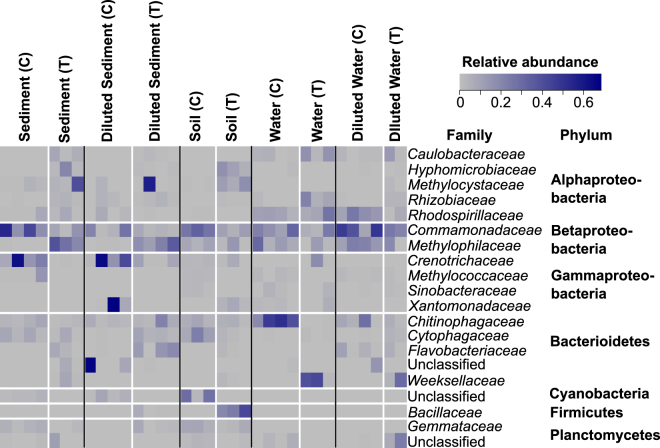
Heatmap of the 20 most abundant non-methanotrophic bacteria at the family and Phylum level derived from the standardized 16S rDNA-based sequencing data (treated water samples n = 3, treated diluted water samples n = 2, treated and control soil samples n = 3, treated sediment samples n = 3, all remaining n = 4). For this analysis standardized 16S rDNA-based sequencing data was further simplified by removing single and doubletons and focusing on sequences with a relative abundance of >1% to obtain a better visual representation. Control (C) and Treatment (T).

We then compared the identity of dominant taxa within methane-consuming communities from perturbated and unperturbed samples (Fig. 4). In particular, the family *Chitinophagaceae* were almost absent in perturbated enrichments originating from water samples. In addition, the families *Methylophilaceae* and *Crenotrichaceae*. displayed strong patterns in enrichments that originated from sediment samples. Members of these families were only found in perturbated samples and were below the detection limit in control samples (Fig. 4). Contrastingly, the family *Commamonadaceae* showed a higher relative abundance in controls than in perturbated samples. This was independent from the origin of samples (Fig. 4).

### Abundance of total bacteria and methanotrophs during a series of dry/wet cycle perturbations

**Figure 5:**
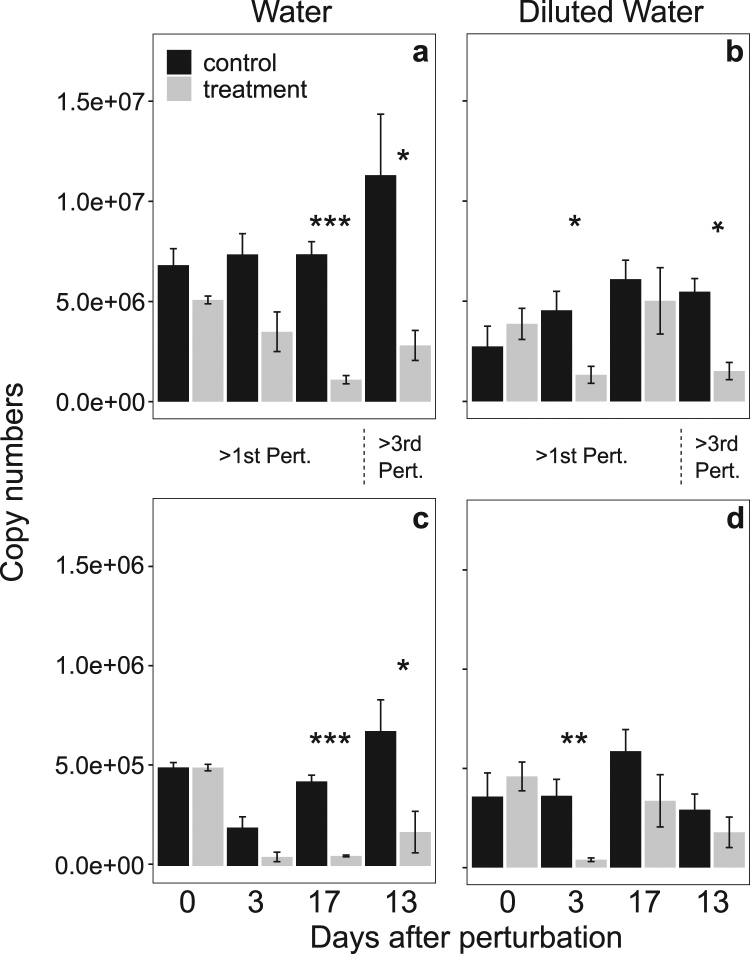
qPCR analysis of total bacteria (based 16S rDNA, **a**,**b**) and total methanotrophic population (based on the marker gene *pmoA*, transcribes subunit of key enzyme in methane oxidation, **c**,**d**) from enrichments originating water samples. The assays were performed in duplicate from each DNA extract (2 ng/μL) for each of the four replicates (mean ± s.d; n = 8) before the first dry/wet cycle perturbation, after three and 17 days of the first dry/wet cycle perturbation (>1st Pert.), and 13 days after the third dry/wet cycle perturbation (>3rd Pert.). Asterisks above the x axis indicate significance between control and treatment conditions at different time points (***P < 0.001; **P < 0.01; *P < 0.05).

Since Illumina 16S rDNA sequencing gives only relative abundances of the present bacterial community we further evaluated temporal dynamics of total bacteria and methanotroph’s abundance using quantitative PCR (qPCR) assays (Fig. 5). We focused on enrichments originating from water samples because these samples depicted clear signs of slowing down in the recovery of methane consumption, which allowed evaluating whether abundance is a driver of loss in resilience. First, we confirmed that the dilution treatment decreased the overall abundance of the bacterial community based on quantitative PCR of the 16S rDNA (Fig. 5). We did not expect total abundance of methanotrophs to vary between dilutions since we enriched for methanotrophs (Fig. 5). Second, the abundance of total and methanotrophic bacteria decreased in disturbed microcosms over time (Fig. 5). We further observed an increase in total and methanotrophic bacteria in the diluted water samples at day 17 after the first perturbation (Fig. 4).

## Discussion

In this study, we used a microbial model system to test legacy effects as basic principle that is important in microbial resilience. We hypothesized that environmental legacy towards drought in enriched and functionally relevant methane-consuming communities originating from soil (always dry), sediment (periodic dry) and water (never dry), persists when exposed to a series of dry/wet cycle perturbations.

In accordance with our hypothesis, we demonstrate that the recovery of methane consumption in enrichments originating from the water column, with no legacy in exposure to dry/wet cycle perturbations, slowed down over time. This indicates a loss of resilience after recurring perturbations, which can prelude a collapse in the methanotrophic community^16^.

Previous work in soil has shown that methane consumption was not considerably compromised by disturbances and even displayed higher activities compared to undisturbed communities, but at the expense of community evenness^24,25^. Hence, one could argue that biomass and changes in community structure by the repeated perturbations are drivers underlying the loss of resilience. However, abundance dynamics of the total bacterial community in enrichments originating from water samples did not fully explain the slowed recovery but suggested a contribution of the non-methanotrophs to the recovery trend (Figs 1 and 5). Similarly, diversity parameters were not directly correlated to the observed differences in recovery but depicted the lowest richness in unperturbed enrichments from water samples (Supplementary Information Table S2). Scheffer and colleagues^26^ suggested that in highly connected (interacting) systems local perturbations can be buffered quickly through feedbacks from the system itself. In this study, soil and sediment enrichments recovered even faster after repeated dry/wet cycle perturbations, given sufficient recovery period (Fig. 2). High interactions between taxa of soil and sediment microbial communities could therefore underlie their fast recovery, even after repeated perturbations.

Previous work already provided direct evidence that the presence of non-methanotrophic heterotrophs resulted in increased methanotrophic activity^27^. Hence, non-methanotrophic heterotrophs may be an important component in the resilience towards perturbations. In this study, we used an experimental design, in which the methanotrophs act as a key species that support non-methanotrophic bacteria, e.g. by cross-feeding carbon from methane to other microbial species^22,23,28^. Members of the family Chitinophagaceae were linked to the dry/wet cycle perturbation in water enrichments, but their effect on methanotrophs remains speculative. Interestingly, the co-enriched members of Beta-proteobacteria were highly abundant in the total bacteria communities and showed distinct patterns between control and perturbated enrichments. Considering methanol as the first product in aerobic methane oxidation it is not surprising that the family *Methylophilaceae*, which includes methanol-utilizers, responded strongly. It suggests that members of the family *Methylophilaceae* may be involved in stabilizing methanotrophic community functioning in enrichments originating from the sediment. By co-enriching methanotrophs and non-methanotrophic bacteria the history of the original habitat may have been preserved in terms of the interacting communities. Hence, a legacy effect may arise from the non-methanotrophic bacteria present originally, co-enriched and supporting methanotrophs in a yet unknown way^29^.

In conclusion, the application of a microcosm model system demonstrates a loss of resilience in functionally relevant methanotrophic microbial communities that have never been exposed to dry-wet cycles. This suggests that legacy effects contribute to the response of microbial processes to perturbation in our study. More mechanistically, we observed an example of slowed recovery of methanotrophic communities giving new support to the existence of slowing down as an indicator before a possible collapse or loss of function. In addition, we fueled the on-going debate that microbial functions widely distributed among microbes are likely to be more redundant and therefore may be compensated by other members of the microbial community^30^ in case of perturbation. Individual taxa may still have important additional functions for specialized groups such as methanotrophs and losses in their diversity will likely reduce important functions such as methane consumption.

## Material and Methods

### Sampling and selective pre-incubations

Soil and water samples were collected in December 2013 from a study area in the Horstermeer polder in the Netherlands, which has previously been described^31–33^. Three cores (20 cm length, 3.8 cm diameter) of soil were taken at random locations with a soil corer. The water column of the ditch was sampled using a bucket and water was transferred into 500 ml jars that were closed with a lid. In addition, three sediment samples were collected in the same way as soil samples from the Polder Nootdorp in the Netherlands, an aquatic system that has been described in a previous study^34^.

Samples were transported back to the laboratory and immediately processed. From the soil and sediment, the top five centimeter were homogenized separately and 5 grams were weighed into 150 ml flasks and 20 ml of five times diluted nitrate mineral salt medium (M2) was added^35^ in triplicates. Flasks were capped with grey rubber stoppers (Sigma-Aldrich, St Louis, MO, USA) and 5% (v/v) pure methane was added. The incubations were performed for one week at 20 °C in the dark on a gyratory shaker (120 rpm) to enrich for methanotrophs and reduce the complexity of the total microbial community to associated functionally relevant non-methanotrophic heterotrophs. Water samples were incubated in the same way as soil and sediment samples except that 5 ml of water were mixed with 15 ml of M2 medium.

### Experimental setup and perturbations

These enrichments of methanotrophic communities from different environments were used as starting material for the main experiment. We prepared two setups to test for the effect of biomass and changes in the total non-methanotrophic community composition to explain loss of resilience. In the first setup enrichments were diluted 1:3000 and in another setup enrichments were used undiluted. For all setup microcosms were prepared that consisted of 120 ml serum bottles that were filled with 20 ml enrichment mix. Enrichment mix was prepared by mixing 20 ml enrichment with 160 ml M2 medium. Microcosms were capped with rubber stoppers (Sigma-Aldrich) and 5% (v/v) pure methane was added to the headspace. The incubations were performed for another week at 20 °C in the dark on a gyratory shaker (120 rpm) to further enrich for methanotrophic communities and to ensure that any initial effects from setting up the experiment were minimized. For each environment we prepared eight microcosms, four replicated controls and treatments, in total 52 samples. Please note that the 8 samples for diluted soil did not show any growth (turbidity) or activity (methane oxidation) before the start of the experimental perturbations and were removed from the experiment.

Three dry/wet cycle perturbations were applied during the experiment, the first after 7 days, a second after 36 days, and a third after 45 days. For each dry/wet cycle perturbation the four treatment microcosms from each environment were dried overnight using pressurized air. Please note that pre-experiments showed that these microcosms will cool down to 15 °C during this process. Therefore, control microcosms that were not dried were incubated at 15 °C without additional methane (at atmospheric levels) during the time of each drying procedure to minimize confounding effects. Afterwards the biomass from dried microcosms was carefully re-suspended in 16 ml MilliQ water to keep salt concentrations similar between treatments and controls and 4 ml of fresh M2 medium were added to both treatments and the controls to minimize nutrient limitation during the experiment. Subsequently, microcosms were incubated as described above until the next perturbation.

Before each dry/wet cycle perturbation 4 ml fresh liquid was taken from all microcosms and spun down in the centrifuge at 20817 × *g*. Cell pellets were stored at −80 °C for further analyses.

### DNA Extraction

Cell pellets taken from all microcosms after 59 days of incubation were used to extract total nucleic acids following the protocol described by^36^ with the following exceptions: We used a modified extraction buffer (112.87 mM Na_2_HPO_4_/7.12 mM NaH_2_PO_4_, pH 7.5; 5% CTAB, 2% SDS, 2% N-Lauroylsarcosine, and 1 M NaCl), frozen cells pellets were added to lysing matrix E tube (MP Biomedicals, Duiven, the Netherlands) and homogenized using the FastPrep®-24 Classic Instrument (MP Biomedicals) for 45 sec at 6.5 m/s, and nucleic acids were precipitated for 90 min at 4 °C by using 2 volumes of 30% PEG 6000 in 1.6 M NaCl. DNA quality and quantity were determined using a Nano-Drop Spectrophotometer (Thermo Scientific, Madison, WI, USA).

### Methane consumption

Methane consumption was measured in each microcosm at the following times: before the first dry/wet cycle perturbation, 1, 3, 6, 17, 36 days after the first dry/wet cycle perturbation, 1 and 5 days after the second dry/wet cycle perturbation, and 1, 5, 13, 23 days after the third dry/wet cycle perturbation. Each time microcosms were opened and aerated before microcosms were re-capped with a butyl rubber stopper (Sigma-Aldrich) and 1% of pure methane was added to the headspace. Microcosms were incubated on a rotary shaker (120 rpm) in the dark at room temperature. Methane consumption was followed by GC-FID analysis (Ultra GC gas chromatograph, Interscience, The Netherlands; Rt-Q-Bond 30 m, 0.32 mm, ID capillary, Restek, USA) over a period of two days, including 5 measurements. Column, injector, and detector temperature was set to 80, 150 and 250 °C, respectively. Helium was used as the carrier gas and hydrogen as burning gas. Methane consumption rates for each concentration per sample were calculated by linear regression using the R version 3.2.5^37^.

### Normalized recovery

We used methane consumption to calculate recovery from the first and third dry/wet cycle perturbation. Therefore, the values obtained from disturbed microcosm were divided by the average values of the control microcosms. Once values recovered to the levels of the control we set these values to one, i.e. full recovery from each perturbation.

### Illumina sequencing and data processing

DNA samples from Day 59 of the experiment were sent for sequencing the V4 region (515F-907 R, Supplementary Information Table S3) of the bacterial 16S rRNA gene using paired-end sequencing (2 × 250 bp) on an Illumina Miseq instruments. PCR and sample preparation has been described in detail in Ren and colleagues^38^.

Sequencing data was processed using the quantitative insights into microbial ecology (QIIME) pipeline^39^ in the standard configuration. In brief, low quality paired end sequence reads were removed (sequence lengths <150 bp and average quality scores <25) and sequences were demultiplexed to assign reads to different samples, resulting in sequences with 395 +/− 5 nucleotides. The data has been archived with the NCBI BioProject (PRJNA421932). The UCLUST method was used for OTU clustering^40^.

Clustering was performed at 97% and chimeras were identified using the ChimeraSlayer reference database^41^. A representative sequence from each OTU was aligned with PyNAST^42^. Taxonomy was assigned using the RDP Classifier from the Ribosomal Database Project downloaded on June 22, 2015^43^.

### qPCR

We performed quantitative PCR (qPCR) to determine abundances of total bacteria and methanotrophs for the water samples before the first dry/wet cycle perturbation and after three, seventeen days after the first dry/wet cycle perturbation, and thirteen days after third dry/wet cycle perturbation. The EUBAC assay was used to quantify the total 16S rDNA gene copies^44^ and the *pmoA*-specific qPCR assays MTOT (total methanotrophs) were used to enumerate methanotrophs^45^. The qPCR assays were performed with primers, primer concentration, and PCR profiles as described by Fierer and colleagues^44^ and Pan and colleagues^46^, respectively. All qPCR assays were performed in duplicates. Specificity of the amplicon was verified by melting curve analysis. All analyses were performed with a Rotor-Gene Q real-time PCR cycler (Qiagen, Venlo, Netherlands). To quantify total copy number of each individual assay the Rotor-Gene Q Series Software (Qiagen) was used. *pmoA* copy numbers were divided by two, which is the average number of this gene in methanotrophic genomes.

### Statistical analysis

Statistical and graphical analyses were performed using R version 3.2.5 and 3.3.2^37^. The OTU table from the Illumina sequencing was first rarefied to 2140 sequences per sample using the *rrarefy* function in the *vegan* package implemented in R. To test for differences in the community structure we used Analysis of similarities (ANOSIM) based on Bray–Curtis dissimilarities in the *vegan* package. Nonmetric dimensional scaling (NMDS) was performed using the *metaMDS* function in the *vegan* package. The dissimilarity matrix (Bray–Curtis) was calculated with the *vegdist* function in the *vegan* package^47^. Heatmaps and graphs were prepared using the *gplots* package^48^. Diversity parameters, richness, evenness, and Shannon diversity were calculated with function *diversity* in the *vegan* package^47^. To evaluate qPCR results we first performed a F-Test, followed by the appropriate T-Test (equal or unequal variance, two-sided).

### Data availability

The datasets generated during and/or analyzed during the current study are available from the corresponding author on reasonable request.

## Electronic supplementary material

Supplementary Information
