# Supplementary material for: Environmental legacy contributes to the resilience of methane consumption in a laboratory microcosm system
Source: Sci Rep. 2018 Jun 11;8:8862. doi: 10.1038/s41598-018-27168-9 (PMC5995846; doi:10.1038/s41598-018-27168-9)
Supplement: Supplementary file 1 — Supplementary Information [file 41598_2018_27168_MOESM1_ESM.doc]

Scientific Reports Supplementary Information for manuscript:

**Environmental legacy contributes to the resilience of methane consumption in a laboratory microcosm system**

by

**Sascha M.B. Krause, Marion Meima Franke, Annelies J. Veraart, Gaidi Ren, Adrian Ho, and Paul L.E. Bodelier**

**Table S1**: Recovery after disturbance (Methane consumption values obtained from disturbed microcosm were divided by the average values of the control microcosms, i.e. recovery compared to the control in percent).

| *Origin* | *Replicate* | *1* | *3* | *6* | *17* | *36* | *1* | *5* | *1* | *5* | *13* | *23* |
| --- | --- | --- | --- | --- | --- | --- | --- | --- | --- | --- | --- | --- |
| *Water* | 1 | 0.00 | 0.00 | 0.00 | 0.92 | 1.17 | 0.00 | 0.00 | 0.00 | 0.00 | 0.00 | 1.11 |
|  | 2 | 0.00 | 0.00 | 0.00 | 0.76 | 1.05 | 0.00 | 0.00 | 0.00 | 0.00 | 0.00 | 1.27 |
|  | 3 | 0.00 | 0.00 | 0.00 | 0.94 | 1.10 | 0.00 | 0.00 | 0.00 | 0.00 | 0.71 | 0.09 |
|  | 4 | 0.00 | 0.00 | 1.07 | 1.03 | 1.30 | 0.00 | 0.00 | 0.00 | 0.00 | 0.00 | 1.08 |
| *Diluted water* | 1 | 0.00 | 0.00 | 0.00 | 0.62 | 1.19 | 0.00 | 0.00 | 0.00 | 0.00 | 0.59 | 1.19 |
|  | 2 | 0.00 | 0.00 | 1.00 | 0.88 | 1.03 | 0.00 | 0.00 | 0.00 | 0.00 | 0.14 | 1.19 |
|  | 3 | 0.00 | 0.00 | 1.14 | 0.96 | 1.40 | 0.06 | 0.00 | 0.00 | 0.00 | 0.00 | 1.34 |
|  | 4 | 0.00 | 0.00 | 0.76 | 0.97 | 1.00 | 0.00 | 0.00 | 0.00 | 0.00 | 0.00 | 1.42 |
| *Soil* | 1 | 0.00 | 0.00 | 0.00 | 1.12 | 1.38 | 0.00 | 0.44 | 0.00 | 0.59 | 0.88 | 1.26 |
|  | 2 | 0.00 | 0.00 | 0.36 | 1.19 | 1.15 | 0.00 | 0.92 | 0.00 | 0.35 | 0.85 | 1.21 |
|  | 3 | 0.00 | 0.00 | 0.00 | 1.23 | 1.12 | 0.00 | 0.27 | 0.00 | 0.65 | 0.93 | 1.15 |
|  | 4 | 0.00 | 0.00 | 0.00 | 1.17 | 1.21 | 0.00 | 0.27 | 0.00 | 0.80 | 0.70 | 1.22 |
| *Sediment* | 1 | 0.00 | 0.00 | 0.00 | 1.02 | 0.45 | 0.10 | 0.66 | 0.00 | 0.42 | 1.11 | 1.05 |
|  | 2 | 0.00 | 0.00 | 0.82 | 1.02 | 1.32 | 0.00 | 1.07 | 0.00 | 0.00 | 1.08 | 1.09 |
|  | 3 | 0.00 | 0.00 | 0.00 | 1.04 | 0.53 | 0.17 | 0.51 | 0.00 | 1.06 | 0.00 | 1.12 |
|  | 4 | 0.00 | 0.00 | 0.00 | 1.01 | 0.74 | 0.20 | 0.44 | 0.00 | 0.41 | 1.08 | 1.06 |
| *Diluted sediment* | 1 | 0.00 | 0.00 | 0.00 | 1.09 | 4.19 | 0.00 | 0.00 | 0.00 | 0.24 | 1.74 | 2.19 |
|  | 2 | 0.00 | 0.00 | 0.39 | 1.13 | 4.19 | 0.05 | 0.00 | 0.00 | 0.13 | 0.52 | 2.21 |
|  | 3 | 0.00 | 0.00 | 0.00 | 1.15 | 3.72 | 0.00 | 0.67 | 0.10 | 1.35 | 1.76 | 2.28 |
|  | 4 | 0.00 | 0.00 | 0.56 | 1.22 | 3.57 | 0.22 | 0.84 | 0.34 | 0.94 | 1.25 | 2.20 |

**Table S2**: Diversity parameters of total bacteria from different samples thirteen days after third disturbance.

| **Origin** | **Setup** | **Richness** | **Evenness** | **Shannon** |
| --- | --- | --- | --- | --- |
| Water | control | 6 ± 2 | 0.42 ± 0.32 | 0.79 ± 0.72 |
|  | treatment | 11 ± 7 | 0.66 ± 0.46 | 1.26 ± 0.85 |
| Diluted water | control | 7 ± 3 | 0.49 ± 0.21 | 0.92 ± 0.48 |
|  | treatment | 9 ± 6 | 0.73 ± 0.24 | 1.33 ± 0.13 |
| Soil | control | 16 ± 3 | 0.74 ± 0.02 | 2.06 ± 0.12 |
|  | treatment | 33 ± 9 | 0.37 ± 0.12 | 1.29 ± 0.51 |
| Sediment | control | 48 ± 15 | 0.58 ± 0.16 | 2.22 ± 0.56 |
|  | treatment | 34 ± 7 | 0.53 ± 0.36 | 1.89 ± 1.35 |
| Diluted sediment | control | 23 ± 13 | 0.19 ± 0.09 | 0.52 ± 0.09 |
|  | treatment | 14 ± 9 | 0.48 ± 0.25 | 1.03 ± 0.44 |

**Table S3**: Miseq primer.

| **Sample** | **Replicate** | **Barcode** | **Forward primer** |
| --- | --- | --- | --- |
| Water treatment | 2 | AGCCAGTCATAC | GTGCCAGCMGCCGCGG |
| Water treatment | 3 | AGCGAACCTGTT | GTGCCAGCMGCCGCGG |
| Water treatment | 4 | GTTTGCTCGAGA | GTGCCAGCMGCCGCGG |
| Water control | 1 | CAAACGCACTAA | GTGCCAGCMGCCGCGG |
| Water control | 2 | GAACAAAGAGCG | GTGCCAGCMGCCGCGG |
| Water control | 3 | GCTAAGTGATGT | GTGCCAGCMGCCGCGG |
| Water control | 4 | AAGGGACAAGTG | GTGCCAGCMGCCGCGG |
| Diluted water treatment | 1 | AGTGTCGATTCG | GTGCCAGCMGCCGCGG |
| Diluted water treatment | 3 | CCTACCATTGTT | GTGCCAGCMGCCGCGG |
| Diluted water control | 1 | GATAACTGTACG | GTGCCAGCMGCCGCGG |
| Diluted water control | 2 | TAAACCTGGACA | GTGCCAGCMGCCGCGG |
| Diluted water control | 3 | CCGAATTGACAA | GTGCCAGCMGCCGCGG |
| Diluted water control | 4 | CTGGCATCTAGC | GTGCCAGCMGCCGCGG |
| Soil treatment | 1 | GGTGGTCGTTCT | GTGCCAGCMGCCGCGG |
| Soil treatment | 2 | ACTATGGGCTAA | GTGCCAGCMGCCGCGG |
| Soil treatment | 3 | GCATTGAGTTCG | GTGCCAGCMGCCGCGG |
| Soil control | 1 | CTATGGTGAACC | GTGCCAGCMGCCGCGG |
| Soil control | 3 | GTATTGGTCAGA | GTGCCAGCMGCCGCGG |
| Soil control | 4 | AGAACCGTCATA | GTGCCAGCMGCCGCGG |
| Sediment treatment | 1 | AACTGGAACCCT | GTGCCAGCMGCCGCGG |
| Sediment treatment | 3 | ACGCTTAACGAC | GTGCCAGCMGCCGCGG |
| Sediment treatment | 4 | AGCTTACCGACC | GTGCCAGCMGCCGCGG |
| Sediment control | 1 | AGGGCTATAGTT | GTGCCAGCMGCCGCGG |
| Sediment control | 2 | TGTCTCGCAAGC | GTGCCAGCMGCCGCGG |
| Sediment control | 3 | CAGCCGCATATC | GTGCCAGCMGCCGCGG |
| Sediment control | 4 | GATACGTTCGCA | GTGCCAGCMGCCGCGG |
| Diluted sediment treatment | 1 | CCAAGATTCGCC | GTGCCAGCMGCCGCGG |
| Diluted sediment treatment | 2 | GAGGCTGATTTA | GTGCCAGCMGCCGCGG |
| Diluted sediment treatment | 3 | GAGTTAGCATCA | GTGCCAGCMGCCGCGG |
| Diluted sediment treatment | 4 | TGTAGTATAGGC | GTGCCAGCMGCCGCGG |
| Diluted sediment control | 1 | CTCACGCAATGC | GTGCCAGCMGCCGCGG |
| Diluted sediment control | 2 | GTCCCGTGAAAT | GTGCCAGCMGCCGCGG |
| Diluted sediment control | 3 | GGACAGTGTATT | GTGCCAGCMGCCGCGG |
| Diluted sediment control | 4 | ACACGACTATAG | GTGCCAGCMGCCGCGG |
